# A Systematic Review of Sleep Measurement in Critically Ill Patients

**DOI:** 10.3389/fneur.2020.542529

**Published:** 2020-11-06

**Authors:** Kathy C. Richards, Yan-yan Wang, Jeehye Jun, Lichuan Ye

**Affiliations:** ^1^University of Texas at Austin School of Nursing, Austin, TX, United States; ^2^West China Hospital, Sichuan University, Chengdu, China; ^3^Department of Biobehavioral Health Science, College of Nursing, University of Illinois at Chicago, Chicago, IL, United States; ^4^School of Nursing, Bouve College of Health Sciences, Northeastern University, Boston, MA, United States

**Keywords:** sleep measurement, critically ill patient, ICU—intensive care unit, polysomnography, actigraphy, sleep questionnaire

## Abstract

**Background:** Clinical trialists and clinicians have used a number of sleep quality measures to determine the outcomes of interventions to improve sleep and ameliorate the neurobehavioral consequences of sleep deprivation in critically ill patients, but findings have not always been consistent. To elucidate the source of these consistencies, an important consideration is responsiveness of existing sleep measures. The purpose of an evaluative measure is to describe a construct of interest in a specific population, and to measure the extent of change in the construct over time. This systematic literature review identified measures of sleep quality in critically ill adults hospitalized in the Intensive Care Unit (ICU), and assessed their measurement properties, strengths and weaknesses, clinical usefulness, and responsiveness. We also recommended modifications, including new technology, that may improve clinical usefulness and responsiveness of the measures in research and practice.

**Methods:** CINAHAL, PubMed/Medline, and Cochrane Library were searched from January 1, 2000 to February 1, 2020 to identify studies that evaluated sleep quality in critically ill patients.

**Results:** Sixty-two studies using polysomnography (PSG) and other electroencephalogram-based methods, actigraphy, clinician observation, or patient perception using questionnaires were identified and evaluated. Key recommendations are: standard criteria are needed for scoring PSG in ICU patients who often have atypical brain waves; studies are too few, samples sizes too small, and study duration too short for recommendations on electroencephalogram-based measures and actigraphy; use the Sleep Observation Tool for clinician observation of sleep; and use the Richards Campbell Sleep Questionnaire to measure patient perception of sleep.

**Conclusions:** Measuring the impact of interventions to prevent sleep deprivation requires reliable and valid sleep measures, and investigators have made good progress developing, testing, and applying these measures in the ICU. We recommend future large, multi-site intervention studies that measure multiple dimensions of sleep, and provide additional evidence on instrument reliability, validity, feasibility and responsiveness. We also encourage testing new technologies to augment existing measures to improve their feasibility and accuracy.

## Introduction

Outcomes following critical illness and discharge from the Intensive Care Unit (ICU) can range from full recovery to varying degrees of disability. There is more and more evidence that sleep deprivation during the ICU stay has both negative short-term effects, such as poor comprehension of discharge instructions and delirium, and lasting serious consequences, such as cognitive impairment, that are of key interest and importance to patients and their families, clinicians, hospitals, and payers (1). Clinical trialists have used several measures of sleep to assess the effectiveness of interventions aimed at improving sleep and ameliorating the consequences of poor sleep on neurobehavioral function. Results have not been always been consistent. To elucidate the source of these consistencies, an important consideration is suitability of sleep measures. Reliable and valid evaluative measures of sleep are required to measure the outcomes of interventions to improve sleep in ICU patients and prevent the negative effects of sleep deprivation.

The purpose of an evaluative measure is to describe a construct of interest in a specific population, and to measure the extent of change in the construct over time. Sleep is a multi-dimensional construct, composed of dimensions such as total sleep time, percent of sleep stages, frequency of awakenings or arousals, expectations, global perceptions, sleep movements, tiredness upon awakening, daytime energy levels, and functional impairments. Various measures of sleep in ICU patients exist, but they do not all measure exactly the same dimensions, and we do not expect them to demonstrate 100% agreement. For example, the Richards Campbell Sleep Questionnaire (RCSQ) (2) measures the dimension of patients' perception of their sleep. Polysomnography, on the other hand, measures a different dimension, objective sleep quality using the electroencephalogram (EEG), electromyogram, and electrooculogram. Objective sleep quality consists not only of the total duration of sleep, but also of the architecture of sleep (amount of different sleep stages) amount of wake, frequency and duration of awakenings, and other factors.

In this systematic literature review we identified measures of sleep in critically ill adults hospitalized in the ICU and discussed the dimensions of sleep that they measure. We focused on publications from 2000 to 2019 because the ICU environment and care delivery has significantly changed, and earlier studies may not be relevant to the current ICU setting. We evaluated the strengths and weaknesses of the measures, and their clinical and research usefulness. As an essential step, we assessed their measurement properties based on the criteria described by McDowell (3) and Jeffs and Darbyshire (4). Three aspects were critiqued: construct validity (i.e., *whether the tool adequately and appropriately evaluated patient sleep?*); criterion validity (i.e., *is this tool comparable or agreeable with other standard measures of sleep?*); and reliability and consistency (i.e., *is there any measure of reliability reported, such as Cohen's kappa for scoring polysomnography, test-retest reliability, or Cronbach's alpha for internal consistency*?). Because choosing an optimal measure of sleep for clinical or research purposes should not be based on measurement properties alone, we also discuss feasibility, and responsiveness. We focused on four measures of sleep quality: polysomnography and other electroencephalogram-based methods, actigraphy, clinician observation, and patient perception using questionnaires. We also recommended modifications of existing sleep measures that may improve their reliability and usefulness in research and practice, including adding innovative new technology.

## Methods

### Search Strategies

A systematic database search was performed in February 2020. We conducted a search on PubMed/MEDLINE, CINAHL, and Cochrane Library with the following combination of MESH terms/ keywords: sleep AND (critical care OR intensive care OR ICU). The inclusion criteria were: (1) primary sources published from 2000 through 2019; (2) systematic or focused reviews 2000–2019, (3) written in English and electronically available in full-text format; and (4) measured sleep in the ICU using at least one method. The authors evaluated the titles and abstracts of all potentially useful studies based on the inclusion criteria and identified articles for a full-text review. Additional relevant studies, such as those referenced by reviews, were further included. The reviewers reached a consensus on which original research studies were to be included in the review. If an article described a measure developed prior to 2000 we reviewed the original publication.

### Data Extraction and Critical Appraisal

**Figure 1:**
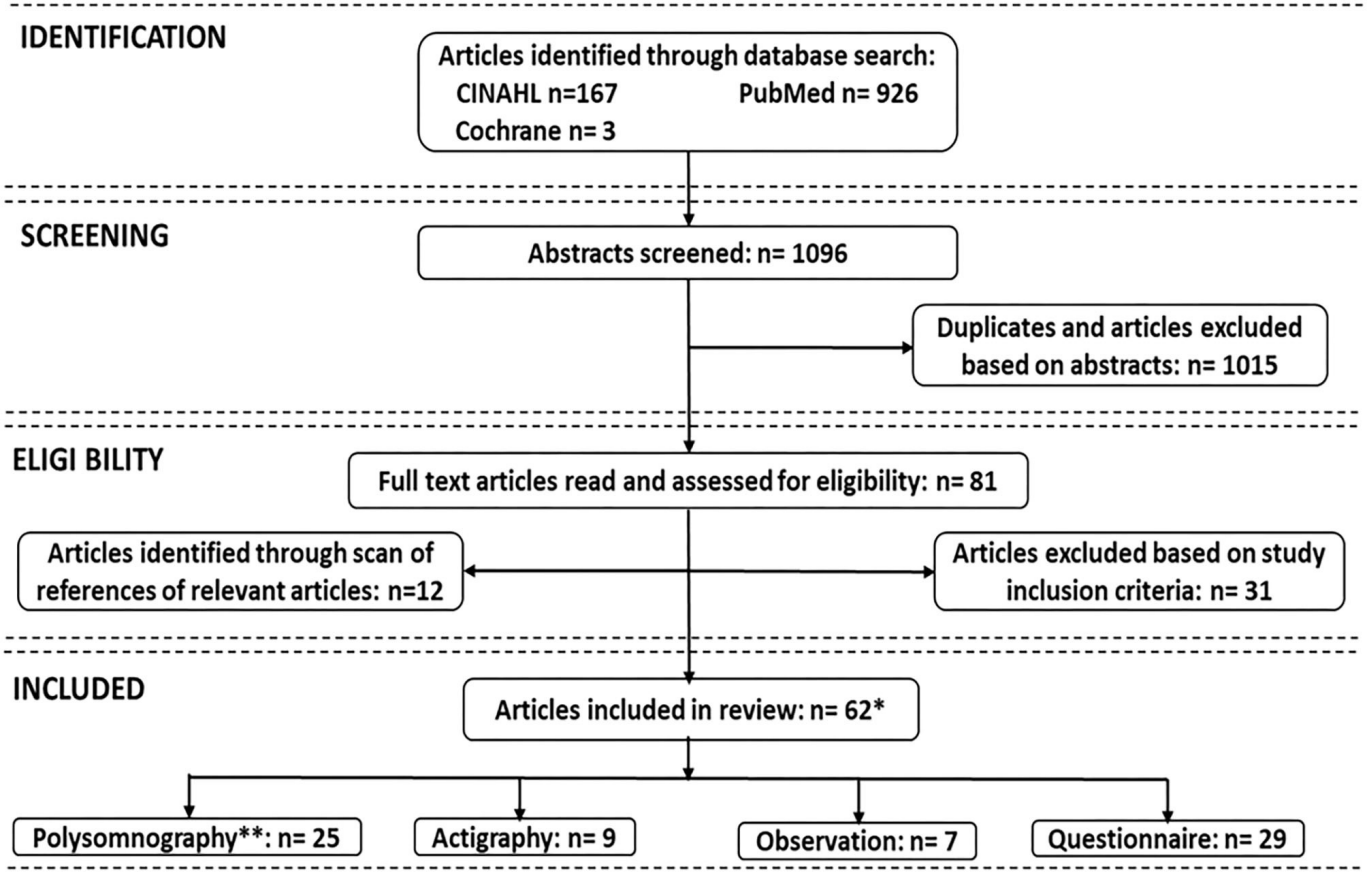
Search strategies for measures of sleep in critically ill patients. **Due to overlaps, some studies used more than one sleep assessment; *Total number is 62.

The electronic database search initially identified 1,096 studies (CINAHL 167, PubMed/Medline 926, Cochrane 3). After removal of duplicates, the titles and abstracts of 1,015 articles were examined, resulting in selection of 81 articles for full-text reading. After exclusion of articles that did not meet the inclusion criteria, and adding articles from reference lists, a total of 62 studies were included in this review. Figure 1 depicts the processes for identifying the articles included. Table 1 identifies the authors and dates of the included literature.

**Table 1:** All included literature by category.

**Polysomnography (*N* = 25)**	**Actigraphy (*N* = 9)**	**Observation (*N* = 7)**	**Questionnaires (*N* = 29)**
Cooper et al. (5)	Raymond et al. (6)	Olson et al. (7)	Richards et al. (2)
Richards et al. (2)	Bourne et al. (8)	Ibrahim et al. (9)	Richards et al. (10)
Freedman et al. (11)	Beecroft et al. (12)	Richardson et al. (13)	Richardson et al. (14)
Parthasarathy and Tobin (15)	Chen et al. (16)	Beecroft et al. (12)	Frisk and Nordström (17)
Gabor et al. (18)	van der Kooi et al. (19)	Dennis et al. (20)	Ugras et al. (21)
Hardin et al. (22)	Hamze et al. (23)	Litton et al. (24)	Richardson et al. (13)
Alexopoulou et al. (25)	Kamdar et al. (26)	Aitken et al. (27)	Toublanc et al. (28)
Ambrogio et al. (29)	Naik et al. (30)		Nicolas et al. (31)
Beecroft et al. (12)	Hsu et al. (32)		Bourne et al. (8)
Drouot et al. (33)			Scotto et al. (34)
Kondili et al. (35)			Li et al. (36)
Gehlbach et al. (37)			Kamdar et al. (1)
Watson et al. (38)			Jones and Dawson (39)
Cordoba-Izquierdo et al. (40)			Van Rompaey et al. (41)
Alexopoulou et al. (42)			Bihari et al. (43)
Su et al. (44)			Little et al. (45)
Elliott et al. (46)			Zhang et al. (47)
Elliott et al. (48)			Elliott et al. (46)
Knauert et al. (49)			Kamdar et al. (50)
Alexopoulou et al. (51)			Su et al. (44)
Vacas et al. (52)			Maidl et al. (53)
Huttmann et al. (54)			Hata et al. (55)
Boyko et al. (56)			Storti et al. (57)
Boyko et al. (58)			Ugras et al. (59)
Demoule et al. (60)			Demoule et al. (60)
			Menear et al. (61)
			Aitken et al. (27)
			Rood et al. (62)
			Louis et al. (63)

## Results

### Polysomnography and Other Electroencephalogram-Based Methods

#### Background

Polysomnography (PSG), a multi-parametric recording of the biophysiological changes based on electroencephalographic (EEG) activity, combined with concurrent polygraphic monitoring of electrooculogram (EOG), electromyogram (EMG), electrocardiogram (ECG), as well as other parameters that occur during sleep, has long been regarded as the gold standard for objectively measuring quality and quantity of sleep for comparatively healthy populations outside of the ICU (64). The sleep assessment methods are divided into five categories (65): Type 1- standard PSG: in-laboratory, technician-attended, overnight recording using a minimum of seven channels, including EEG, EOG, submental EMG, ECG, oronasal airflow, respiratory movement, and oxyhemoglobin saturation; Type 2- comprehensive portable PSG: recording using a minimum of seven physiological channels, as in standard PSG, but performed in an unattended setting; Type 3—modified portable obstructive sleep apnea (OSA) testing in an attended setting (also referred to as cardiorespiratory sleep studies): recording of at least four channels (respiratory movement, airflow, heart rate or an electrocardiogram, and arterial oxygen saturation); Type 4—portable, continuous single or dual bioparameter devices, typically recording arterial oxygen saturation and airflow, can be used without a technician for diagnosing OSA.

In addition to the 4 types of multi-parametric devices, alternative portable brain function monitors involve the use of processed EEG, such as the Bispectral Index (BIS) (66), and SedLine^@^ Brain Function Monitor (67).

The original Rechtschaffen and Kales sleep scoring manual (R&K rules) (68) was used until 2007, at which point the American Academy of Sleep Medicine (AASM) updated the scoring manual, known as the AASM scoring manual (69). The R&K method divided sleep into five distinct stages: non-rapid eye movement [non-REM (NREM)] stages 1, 2, 3, and 4 and stage REM sleep. The AASM scoring manual redefined four sleep stages: Stage N1 (formerly stage 1 sleep), stage N2 (formerly stage 2 sleep), stage N3 (formerly stages 3 and 4 sleep), and stage R sleep (formerly stage REM sleep). In addition, the AASM criteria specified different EEG electrodes (R&K used central EEG leads, and AASM used frontal, central, and occipital EEG leads) for recording sleep. The AASM criteria also specified different scoring rules for slow wave sleep (R&K consisted of both stage 3 and stage 4 sleep with delta wave amplitude using central leads vs. the AASM stage N3 sleep criteria for delta wave amplitude using frontal leads). There also were differences in the manuals regarding stage N2 sleep (3-min rule used in R&K, but not in AASM), and whether major body movements impacted scoring.

The R&K and AASM criteria were developed for recording and scoring sleep in typical healthy individuals, without neuropathology or psychoactive medication use, in the controlled environment of a sleep laboratory or the usual home sleep environment. Applying these standard criteria for recording and scoring sleep to critically ill patients is challenging. Typical ICU environments are noisy, and treatments often are invasive and intensive. Sedatives, analgesics, the stress response, mechanical ventilation, and neuropathology may result in atypical brain waves, muscle tension, eye and body movements. Multiple illness-related factors often are associated with atypical biophysiological sleep activity, e.g., sleep fragmentation with frequent arousals and awakenings, disorganized circadian rhythms, and disrupted sleep architecture (increased stage N1 and N2 sleep, and decreased stage N3 and REM sleep) (5, 11). Atypical polysomnographic findings, including lack of the N2 EEG markers *K* complexes and sleep spindles, the presence of polymorphic delta, burst suppression, and isoelectric electroencephalography (5, 11, 38) challenge conventional sleep-scoring rules. Other obstacles to PSG feasibility in ICU setting include large amounts of technician time, concerns about lead displacement, and electrical interference (70, 71).

#### Results

Twenty-five studies (2, 5, 11, 12, 15, 18, 22, 25, 29, 33, 35, 37, 38, 40, 42, 44, 46, 48, 49, 51, 52, 54, 56, 58, 60) using PSG were included in this review, and 17 (68%) were observational. The sample sizes ranged from 8 to 70, with a total of 685 ICU patients included. The most common patient diagnoses were acute respiratory failure, acute coronary syndrome, acute kidney injury, and sepsis. Most of the patients (454/685, 66%) were on mechanical ventilation, either with or without sedation.

Most of the included studies used portable, unattended PSG for at least 8 consecutive hours, and only about half of the studies (13/25) used PSG ≥24 h. Five studies (2, 12, 37, 42, 51) were continuously or periodically attended by a sleep expert or trained research assistant. Total sleep time (TST), sleep efficiency (SE), arousals and awakenings, sleep fragmentation, and sleep architecture (% time of NREM 1, NREM 2, SWS, and REM) were often reported. Six studies (2, 12, 44, 46, 48, 60) concurrently used subjective measures along with PSG to comprehensively assess sleep quality, including behavioral assessment by nurses, the Richards Campbell Sleep Questionnaire (RCSQ), the Sleep in Intensive Care Questionnaire (SICQ), self-assessment of sleep quality, and the Verran/Snyder-Halpern (VSH) sleep scale (72).

Over half (15/25) of the studies used standard sleep scoring (R&K or AASM). Although the R&K method showed good to excellent interobserver reliability for assessing sleep in ambulatory individuals (Cohen κ range 0.68–0.82) (73, 74), the absence of *K* complexes or sleep spindles in 20–44% of ICU patients (5, 11, 29), and the expected decrease in amplitude of delta waves associated with aging renders R&K less useful in the classification of sleep stages in ICU patients, especially for stages N1 and N2 sleep. Hardin et al. used a modified delta (mDelta) criteria which consisted of a frequency criterion of 4 Hz and an amplitude criterion of >50 μV (peak to peak) instead of the standard 75-μV criteria (22). Ambrogio et al. reported that the interobserver reliability of R&K is poor for scoring stage 1 and 2 sleep (κ = 0.19) in critically ill patients, but with better agreements for scoring REM sleep (κ = 0.70) (29). In their study, they used an automated computer-based method of spectral analysis of EEG signals with Fast Fourier Transform (FFT) that showed 100% interobserver agreement for analyzing sleep in critically ill patients, which was significantly better than visual methods using R&K. Gehlbach et al. (37) further assessed sleep EEG and circadian rhythmicity simultaneously over 24 h to determine whether normal sleep organization was detectable in acutely ill patients receiving mechanical ventilation and intravenous sedation. In addition to conventional EEG power spectral analysis, they also calculated the spectral edge frequency 95% (SEF95) for each 30-s epoch to minimize the contribution of residual high-frequency power due to artifacts. SEF95 is defined as the frequency below which 95% of the spectral power resides, with lower SEF95 indicating sleep and higher values indicating wakefulness. They failed to detect normal characteristics of sleep in the special population either by expert visual sleep scoring or by spectral analysis.

In another study, Drouot et al. (33) extended Ambrogio's work by combining EEG spectral analysis and visual quantitative EEG analysis to develop a new classification for sleep analysis in mechanically ventilated, non-sedated, and conscious ICU patients (*N* = 57]. The Drouot group found that sleep cannot be classified with standard criteria in almost 1/3 of the ICU population (16/57, 28%) who were non-sedated conscious patients with respiratory failure requiring non-invasive ventilation (NIV, *N* = 27, 8 atypical sleep) or mechanical ventilation (MV, *N* = 30, 8 atypical sleep). Atypical sleep was characterized by prolonged periods of high-amplitude (50–100 μV), continuous, irregular delta activity without superimposed fast frequencies or rapid eye movements, and with a low submental muscle tone. Further, they suggested that 2 new states of sleep, atypical sleep (an atypical EEG pattern during sleep) and pathologic wakefulness (slow EEG activity during wake), should be added. They proposed a method to identify these 2 new states that involves visually examining the background EEG rhythm in the occipital channel while the patient is awake with eyes closed, followed by examining the patient's EEG reactivity to eye opening and the peak EEG frequency using spectral analysis. By using this approach, atypical sleep was predicted with a sensitivity of 100% and a specificity of 97% in non-sedated conscious ICU patients (33).

On the basis of Drouot's work and Young's EEG classification (75) for coma, Watson et al. (38) further defined the characteristics of atypical sleep in severely ill patients either off mechanical ventilation or on mechanical ventilation with light levels of sedation. The Watson team provided a standardized method to track EEG evidence of pathological brain states, and the effects of sedatives and analgesics which achieved a high interrater reliability (weighted κ = 0.80 [0.48, 0.89]) (38). This novel new scoring method combines behavioral observational assessments necessary for determining wakefulness with EEG analysis, which is a strength. Further studies may consider combining stages (6 stages may be burdensome for scorers), larger sample sizes, and including participants with additional comorbidities, metabolic disturbances, and more sedation. Table 2 summarizes proposed scoring criteria for sleep in critically ill patients.

**Table 2:** Scoring polysomnography and EEG-derived data for sleep in critically ill patients.

**Methods/sample**	**Rationale/background**	**Description**	**Performance/recommendations**
Modified delta (mDelta) criteria (22) 18 mechanically ventilated ICU patients, mean age 54.0 yrs, sd13.0, with IS, CS, or CS with NMBA	• Decreased amplitude of delta waves associated with aging. • Scoring with traditional R&K criteria may underestimate SWS in older adults.	mDelta criteria consisted of a frequency criterion of <4 Hz and an amplitude criterion of >50 μV (peak to peak).	Compared to published normal values, all chemically paralyzed patients in this study had increased delta activity, whether scoring was traditional R&K or mDelta. There was no statistical difference in percent of SWS between traditional R&K and mDelta scoring, and no apparent benefit for using mDelta scoring in chemically paralyzed critically ill patients.
Spectral analysis (29) 14 mechanically ventilated ICU patients and 17 age-matched ambulatory controls, mean age 66.0 yrs, sd 10.0 and 64.0 yrs, sd 11.0, respectively	• Spectral analysis is an automated method that quantifies EEG activity across the EEG spectrum. • Excellent reliability in normal subjects, but untested in critically ill patients	Sleep scoring by 3 manual methods; (1) R&K, (2) sleep-wake organization pattern, and (3) visual detection of burst suppression; and 1 computer-based method: spectral analysis of EEG signals with FFT	Reproducibility for spectral analysis was better than manual methods (R&K and sleep-wake organization pattern) (*P* = 0.03). The intraobserver and interobserver agreement of relative proportions of δ, θ, α, and β power were perfect (κ = 1.0) for critically ill patients. • Overall interobserver reliability for R&K was poor (κ = 0.19) in critically ill patients. Poor interobserver agreement for stage N1 and N2 sleep (κ = 0.01 and 0.18), fair agreement for SWS and wakefulness (both κ = 0.21), and good agreement for REM sleep (κ = 0.7). • Recommend additional research comparing FFT and manual scoring in critically ill patients. • Recommend that publications in ICU patients should report interrater and intrarater reliability of scoring by stage. Investigators should set thresholds for reliability, and retest at specified intervals.
Atypical sleep and pathologic wakefulness (33) 57 non-sedated conscious patients receiving NIV or MV, aged 58–85 yrs	Sleep cannot be classified with standard criteria in one third of mechanically ventilated, non-sedated, and conscious ICU patients.	• To add 2 new states: atypical sleep and pathologic wakefulness; Quantitative assessment of sleep/wake EEG patterns: EEG peak frequency, EEG reactivity, EEG power spectra. • This method visually examines the background EEG rhythm in the occipital channel while the patient is awake with eyes closed followed by the patient's EEG reactivity to eye opening plus the peak EEG frequency using spectral analysis.	Atypical sleep was predicted with a sensitivity of 100% and a specificity of 97% in non-sedated conscious ICU patients by using this method.
Revised scoring system incorporating frequently seen atypical characteristics (38)	• Pathologic wakefulness: any EEG frequency other than alpha or beta with behavioral characteristics of wakefulness. • At1 (Atypical 1): alpha and/or theta present on >10% of epoch, without sleep spindles or K-complexes in the preceding 3 min; may have polymorphic delta, FIRDA, or triphasic activity. • At2: Polymorphic delta, FIRDA, or triphasic activity with alpha or beta activity superimposed on delta waves, without sleep spindles or K-complexes in the preceding 3 min. • At3: Polymorphic delta, FIRDA, or triphasic activity without alpha or beta activity superimposed on delta waves. • At4: Burst-suppression pattern with EEG amplitude <5 μV for >0.5 s. • At5: Suppressed pattern with EEG amplitude <20 μV. • At6: Isoelectric activity (amplitude <5 μV) throughout epoch.		High interrater reliability (weighted κ = 0.80 [0.48,0.89])

*CS, continuous sedation; FFT, Fast Fourier Transform; IS, intermittent sedation; NIV, non-invasive ventilation; NMBA, neuromuscular blocking agents; MV, mechanical ventilation; R&K, Rechtschaffen and Kales; SWS, slow wave sleep; EEG, electroencephalogram*.

A number of processed EEG-based brain function monitors were originally developed to monitor sedation during anesthesia, and some have undergone limited testing as potential measures of the sleep/wake state in critically ill patients. An advantage of most of these monitors in the ICU, vs. PSG, is that a technician does not need to be in attendance to ensure a good recording, and replacement of sensors do not require a skilled technologist as with PSG (70). However, similar to traditional EEG, brain function monitors are subject to electrical interference and increased EMG activity may affect signal quality. Additional concerns are lack of validated scoring rules, and lack of studies of sensitivity and specificity. One measure, the Bispectral Index (BIS), is calculated from multiple analyses of the raw EEG waveform, including power spectral analysis, bispectral analysis, and time-based analysis. Overall, BIS values near 100 represent an awake state, and BIS values fall during physiological sleep, but there is significant overlap of values for a given sleep stage (70). Nicholson et al. (76) used BIS and submental EMG as an indicator of sleep patterns in 27 recovering ICU patients (mean age 64 years, range 15–82 years). Sleep was measured overnight, from 10 p.m. to 8 a.m. Sleep classifications were: Wake—BIS >85; Light Sleep—BIS 60–85; Slow Wave Sleep—BIS <60; and REM—BIS >60 and either a decrease in EMG power >30% or the presence of REM-like waves on the frontal EEG. They found that no ICU patient showed a completely normal sleep pattern (12 of the patients were classified as having a CYCLICAL sleep pattern, 3 had no sleep, and 12 showed ABNORMAL patterns). Many of the ABNORMAL group appeared to be in a REM-like state—having a high BIS but a low level of consciousness (76). Vacas et al. (52) tested another brain monitor, SedLine, against PSG in 3 in-laboratory, primarily healthy subjects. Agreement between SedLine and in-laboratory PSG was good, with 75% overall agreement, 67% for wake, 77% for stage NREM, and 89% for stage REM. However, agreement between the SedLine and PSG was much less for sleep stages, only 29% for N1 and 6% for N3. The Vacas group then tested the feasibility of the BIS in 23 ICU patients. The mean recording time per patient was 19.1 h, and they found that the device was feasible to measure sleep and wake EEG data without interfering with nursing care and patient management. In summary, processed EEG- based brain function monitors have potential as measures of sleep in ICU patients in the future, but additional high-quality ICU studies are needed that correlate processed EEG with sleep, wake, and sleep stages measured by PSG.

#### Summary of Strengths, Weaknesses, and Recommendations

Polysomnography remains the gold standard for evaluating physiological sleep in ICU patients. However, there are a number of challenges, such as technical difficulties (placement and maintenance of electrodes, data interpretation), acceptability by patients, family, and clinical staff (i.e., patients' discomfort, severity of illness and ventilator status, and interference of complex treatment and patient transfer, etc.), as well as additional expense. The greatest challenge to date has been lack of reliability for scoring sleep due to atypical EEG findings often found in ICU patients. Recently, several investigators have addressed this challenge by developing and validating ICU specific scoring rules. We recommend that investigators should always report PSG recording and scoring methods and justify their choices. Compared to PSG, other portable EEG-based monitors are more feasible in ICU patients, but their validity as alternatives to PSG in the ICU setting require further testing.

### Actigraphy

#### Background

The actigraph is a motion sensor detector (accelerometer) similar in size to a wristwatch that is used to assess motor activity. The device can be used to determine physiological sleep or waking during each set epoch by counting activity within a defined threshold (12). A sleep algorithm generally includes total sleep time (hours), wake after sleep onset (minutes), onset latency (minutes), sleep latency (minutes), sleep efficiency (percent), and number of awakenings. An actigraph is typically placed on the wrist or ankle of a patient while avoiding any medical instruments present. Recent clinical practice guidelines cite numerous studies on the validity of actigraphy, and the guidelines conclude that actigraphy can provide useful metrics across a variety of sleep-wake disorders to assist in assessment and monitoring of treatment response (77). However, actigraphy measures sleep by quantifying movement, and ICU patients have reduced movement due to sedation, bedrest, and monitoring devices, which may limit its usefulness in ICU patients.

#### Results

Relatively few investigators have studied the measurement attributes of actigraphy in critically ill patients. We found nine studies: (6, 8, 12, 16, 19, 23, 26, 30, 32), and six of the studies (67%) were non-experimental (6, 12, 19, 23, 26, 30). Their sample sizes ranged from 7 to 85, and a total of 282 patients were included. Four focused on specific populations—mechanically ventilated (12), post-operative (19), tracheostomized (8), and burn (6) patients—and the other five included general ICU patients (16, 23, 26, 30, 32). Measurement periods ranged from one night to the duration of the ICU stay, and seven studies (78%) described measurement qualities (8, 12, 16, 19, 23, 26, 30). In six of the nine studies, the actigraph was applied on the wrist or arm (6, 12, 19, 23, 30, 32), in one it was placed on either the wrist or ankle (16), and in another it was placed on both the wrist and ankle for data comparison (26). One article did not specify the placement (8). Seven studies used various combinations of sleep quality methods (6, 8, 12, 16, 19, 30, 32), and five of those studies evaluated actigraphy in comparison with other sleep measures (8, 12, 16, 19, 32).

Beecroft et al. (12) studied 12 stable, mechanically-ventilated patients (median age 68 years, IQR = 13). Exclusions were co-morbid disease that could confound interpretation of sleep including neurological disease, sedation, or paralyzing medication. Wrist actigraphy, attended PSG, and nurse assessment were employed for one night from 11 p.m. to 6 a.m. Various actigraph analysis settings for activity threshold and automatic sensitivity were used (30 s epochs). PSG data were recorded and scored by registered PSG technologists using R&K criteria (68). Median sensitivity (epochs correctly scored as sleep) ranged from 43 to 44%, specificity (epochs correctly scored as wake) from 75 to 95%, and accuracy (percentage of epochs scored correctly) from 51 to 61%, with the best accuracy (61%) on the high sensitivity threshold. Actigraphy overestimated total sleep time (actigraphy 5.73 h, IQR 2.64, vs. PSG 3.1 h, IQR 3.26), sleep efficiency (actigraphy 78.1%, IQR = 33.53 vs. PSG 41.90%, IQR 48.55), and the number of awakening (actigraphy 58.50, IQR = 48.00 vs. PSG 40.00, IQR = 74.25) compared to PSG. This was a well-conducted study, with only a few limitations–it had a small sample size with only one night of sleep, and the blinding and interrater reliability of those scoring PSG was not provided.

In another study, van der Kooi et al. (19) examined sleep quality in seven post cardiothoracic surgery patients (median age 65, IQR = 62–72). PSG data were scored according to the AASM guidelines (69) by an experienced technologist who was blinded to actigraphy results, and actigraphy data were automatically scored using proprietary software. Median total recording time was 974 min (IQR 845–1,080). The median sensitivity (the percentage of actigraphy epochs that agreed with PSG for sleep) was 94%. However, the median specificity for detecting wake was only 19%, and surprisingly the number of awakening was significantly correlated with PSG (*r* = 0.76, *p* = 0.049). Limitations of this study were very small sample size, and no discussion of whether alterations were needed or made to PSG scoring criteria.

A recently published article concluded that actigraphy is an objective and relatively reliable measure compared to nurse observations (32). Hsu et al. (32) randomly assigned 60 medical ICU patients (mean age 62.4 years, SD 11.8) to a back massage or a usual care control condition. They measured sleep quality using actigraphy, the VSH sleep scale (72), and nurse assessment during three consecutive nights. The mean of nurse observations of total sleep time was 4.0 (SD = 0.6) hours compared to 5.9 (SD = 0.7) hours with actigraphy. In this study, total sleep time measured by nurses was about 0.6 times lower than actigraphy measurements.

An actigraph is usually applied to the wrist, but the ankle can be used. Kamdar et al. (26) compared actigraphy at the wrist and ankle in 34 patients during 48 h. The mean hours slept by wrist actigraphy was 33.4 (SD = 8.8) hours and 19.6 (SD = 17.2) movements per 30-s epoch, while ankle actigraphy recorded 43.2 (SD = 4.1) hours of sleep and 5.1 (SD = 6.0) movements per 30-s. The authors concluded that actigraphy is feasible and generally well-tolerated in critically ill patients, and that wrist and ankle actigraphy measurements of sleep agree poorly and cannot be used interchangeably.

Three experimental studies (8, 16, 32) measured sleep quality using actigraphy and evaluated intervention effects. Two of the three studies showed improvements in the expected direction, and consistency of treatment response between actigraphy and other sleep measures. In the largest intervention study using actigraphy as an outcome conducted in ICU patients to date, Chen et al. (16) applied 2.5% valerian essential oils and administered valerian acupressure in the experimental group (*n* = 41) for 3 nights, while the control group (*n* = 44) received regular treatment. Post-intervention actigraphy in the experimental group showed a significant increase in sleep hours and a reduction in waking minutes and waking frequency compared to the control group, controlling for baseline. Nurse observed hours slept also showed significant improvement. Table 3 summarizes actigraphy measurement in ICU patients.

**Table 3:** Actigraphy in critically ill patients: design, sample, performance, feasibility, responsiveness, and recommendations.

**References**	**Research design sample**	**Measures where actigraph worn wear time**	**Performance (validity, reliability, accuracy, sensitivity, and specificity)**	**Responsiveness**	**Comments/recommendations**
Raymond et al. (6)	Observational Burn ICU (non-ventilated) *n* = 16	MicroMini Motionlogger Actigraph (Ambulatory Monitoring Inc.). 1-min epochs, Hi-PIM Patient questionnaire Wrist During hospital (14 days on average)	Actigraphy total sleep time mean = 332 min (sd 105) and # awakenings mean = 25.8 (sd 9.5) vs. questionnaire total sleep time mean = 391 min (sd 142) and # awakenings mean = 3.8 (sd 7.5)	N/A	Actigraphy underestimated time slept and overestimated awakenings compared to patient questionnaire. Use specific software for low activity and increase wear time.
Bourne et al. (8)	RCT ICU (Tracheosto-mized) *n* = 24	Actiwatch (Cambridge Neurotechnology) BSI XP, Quattro sensor (Aspect Medical Systems), sleep defined as BSI <80 RCSQ Hourly nurse observations No data on where actigraph worn 4 nights	Placebo group SEI actigraphy = 0.75, BSI = 0.26, RCSQ = 0.50, nurse observation = 0.50 Melatonin group SEI actigraphy = 0.73, BSI = 0.39, RCSQ = 0.41, nurse observation = 0.45	Not responsive—no between group differences in SEI in melatonin group vs. placebo group, BSI difference, but NS (*p* = 0.09) between groups	Actigraphy overestimated sleep efficiency, compared to other measures.
Beecroft et al. (12)	Observational Medical-surgical ICU, on mechanical ventilation *n* = 12	Actiwatch Model AW-64 (Mini Mitter) PSG Nurse assessment Wrist 1 night	Actigraphy analysis conducted using 4 different activity count thresholds Best accuracy (% scored correctly as wake or sleep) was the setting with low activity count threshold for wake (High Threshold mode) Sensitivity 44 (IQR 60), Specificity 86 (IQR 47), Accuracy 61 (IQR 32)	N/A	Actigraphy overestimated total sleep time and sleep efficiency compared to PSG Limitations—only 1 night of actigraphy data, sample size = 12 Recording and scoring criteria R&K, unclear if scoring rules were adapted for abnormal EEG
Chen et al. (16)	RCT (valerian acupressure vs. usual care) ICU patients *n* = 85	Actigraph GT1M, ActiGraph, LLC, ActiWeb software Nurse observation 5 min each hour Wrist or ankle 3 nights	Large differences between TST with actigraphy vs. observation; for example, 2.3 h TST by observation vs. 7.3 h by actigraphy at baseline Nurse observers received sleep observation training, used eye and body movements to determine sleep or wake, and had validity and reliability assessments.	Responsive. Actigraphy showed a significant within group increase in TST and a reduction in wake minutes in the experimental group.	Actigraphy may have overestimated sleep time, as 7.3 h slept is higher than most other studies. The sleep observers monitored patients' sleep for only 5 min every hour. Recommend reducing the intervals between observations.
van der Kooi et al. (19)	Observational Cardiothoracic ICU *n* = 7	Actiwatch (Cambridge Neuro-technology) PSG Wrist Mean duration 974 min (IQR 845–1,080)	The median sensitivity of actigraphy to detect sleep was 94%; median specificity for detection of awakenings was 19%	N/A	Limitations—sample size (*n* = 7), recordings within 3 h of perioperative period, patients were receiving sedation, no discussion of modified PSG scoring criteria for abnormal EEG, actigraph sleep/wake variables not reported
Hamze et al. (23)	Descriptive ICU, *n* = 12	Actisleep (Actigraph Corporation), Actilife version 5 software Wrist 24 h	529 care interventions were recorded by the nurses, but only 21 awakenings were scored by the actigraph. Specificity of the actigraph for detecting wake low.	N/A	The actigraph had a transparent film over it that may have affected results.
Kamdar et al. (26)	Prospective observational Medical ICU *n* = 34	Actiwatch Spectrum (Philips Respironics) Wrist and ankle 48 h	0 movement in 83% of epochs (ankle) and 64% of epochs (wrist); likely overestimated sleep Wrist sleep−33.4 h (sd 8.8); Ankle sleep 43.2 h (sd 4.1)	N/A	Sleep differed based on placement. ICU specific software needed because of low movement of ICU patients; software requires validation. Use wrist; future studies should report sedation, activity, movement restriction.
Naik et al. (30)	Cross-sectional Medical ICU *n* = 32	Actigraph SOMNOwatch (SOMNOmedics GMbH) RCSQ Arm 72 h	Mean TST actigraphy 6.3 h (sd 1.7), RCSQ = 51.6 (sd 13.5)	N/A	ICU TST higher than other studies, actigraphy may have overestimated sleep, also younger subjects in this study (mean = 36.8 years, sd 12.7)
Hsu et al. (32)	Experimental with back massage vs. usual care Medical ICU *n* = 60	Actiwatch 2 (Philips Respironics) VSH Sleep Scale Nurse observations, hourly, IRR among nurse observers = 0.91 Wrist 3 consecutive nights	Actigraph TST = 5.9 h Nurse observation TST = 4.0 h	Responsive. Sleep significantly improved on the 2nd and 3rd days in the intervention group compared to the control group by actigraphy, VSH Sleep Scale, and nurse observation	Limitations: no data on sedatives or correlation of actigraphy, VSH, and observation measures

*Collecting data using actigraphy was feasible in all studies, with little/no missing data*.

*BSI, Bispectral Index; ICU, Intensive Care Unit; IRR, interrater reliability; PIM, Proportional Integrating Mode; PSG, polysomnography; R&K, Rechschaffen and Kales; RCSQ, Richards Campbell Sleep Questionnaire; RCT, randomized controlled trial; SEI, sleep efficiency index; SSS, Standford Sleepiness Scale; TST, total sleep time; VSH, Verran Synder-Halpern Sleep Scale*.

#### Summary of Strengths, Weaknesses, and Recommendations

An actigraph is a non-invasive device used to measure objective sleep quality and has been regarded as an acceptable substitute for PSG due to its lower cost and user-friendliness. Actigraphy is easier to tolerate than multiple PSG leads and provides objective data that is somewhat consistent with PSG. In addition, actigraphy shows moderate responsiveness to interventions as evidenced by improvement in the expected direction and consistency of response with other outcome measures in two of the three clinical trials. The primary weakness of actigraphy is that sleep/wake determination is based on movement, or lack thereof, and ICU patients have reduced movement regardless of their sleep-wake status.

In general, in critically ill patients, actigraphy tends to show higher total sleep time, better sleep efficiency, and more nighttime awakenings compared with PSG, and more overall awakenings compared to nurse assessment and patient questionnaires. However, we identified only nine published articles during the past 20 years, and only five of the nine studies evaluated measurement properties. In general sample sizes were small, and often data were collected for only one night. Also, important information was often lacking, such as PSG scoring reliability and method for dealing with atypical EEG waveforms. Up to 1/3 of PSG data is unable to be reliably scored using standard criteria. Therefore, research on actigraphy in critically ill patients is needed with larger sample sizes, longer durations, and specific sensors and software tested for critically ill individuals who often have low mobility states and often receive sedatives and analgesics. If PSG is used as the comparison, investigators should provide detailed discussion on how atypical EEG waveforms were scored and reliability of scoring. In addition, the ICU population is quite heterogeneous, and the exclusion criteria for reliable actigraphy requires further discussion and consensus. In general, specificity for identifying wake in actigraphy is lower than expected, and sedation, analgesia, and immobility are likely to influence specificity.

### Clinician Observed Sleep

#### Background

Structured observation, also known as systematic observation, is a method for collecting data in which researchers (or clinicians) gather data without direct involvement of participants. Coding of the data is done using previously determined specific behavioral actions. Specific criteria for the behaviors are developed and validated. Data are most often collected by clinicians or research assistants, who have been trained and verified as competent to identify the behaviors. Interrater reliability (consistency between data collectors on coding the behavior) is important, especially in a setting where multiple clinicians or researchers collect data. The observations may be continuous over a specified period of time or completed at specific intervals. Sometimes the data are captured via video, and later scored or coded by humans, or more recently by technology, using specific criteria. Structured observational measures have been used extensively in other populations, for example non-verbal children and older adults with dementia, to measure or identify various behaviors, such as pain.

A few clinician observation sleep tools have been developed and used to identify sleep in ICU patients. These tools identify sleep by structured observations conducted by staff nurses, often in the course of their clinical care, or the tools are used to collect information on patient's sleep retrospectively from the clinicians.

#### Results

In this review we located 7 studies that used a clinician observed sleep measures (7, 9, 12, 13, 20, 24, 27), with 3 (43%) using an experimental design (7, 20). Sample sizes ranged from 12 to 539, and a total of 1,105 patients were included in the 7 studies. Some studies focused on specific patient populations, mechanically ventilated (12), neuro ICU (20), or tracheostomized (9). The observations of sleep were combined with sound levels (24), actigraphy and polysomnography (12), and patient assessments (13, 27). Observational measures used were the Sleep Observation Tool (SOT) (7, 20, 24), and research team developed tools (9, 12, 13, 27).

The SOT, developed and validated by Edwards and Schuring, was designed for nurses in the ICU to assess patient's sleep and wake states at 15-min intervals (78). The nurses mark each patient as asleep, awake, could not tell, or no time to observe following a 5-s observation. During a 4-h data collection period with a total of 340 observations, nurses' assessments of patients' sleep and wake states using the SOT every 15 min had an 81.9% agreement with the PSG-identified sleep-wake status (78). Other researcher-developed observational tools either did not report reliability or validity (9, 27), or were found to be poorly correlated with other measures of sleep (12, 13).

The SOT was used by Litton and colleagues to assess sleep disruption in the ICU in a large prospective multi-site observational study (*n* = 538) (24), and as the outcome measure in two intervention studies, one by Dennis et al. (20) and the other by Olson et al. (7). Both studies examined the effect of an enforced quiet time on sleep. The original 15-min observation interval of the SOT was used by Litton and group to document patient's sleep state from 8 p.m. to 8 a.m. (24). In the two intervention studies the observation intervals were changed to 30-min (7, 20) and patient's sleep during both day and night was documented by nurses caring for the patient. The nurses were, by necessity, unblinded to the quiet time intervention conditions. Patients in both studies were significantly more likely be asleep, as measured by the SOT, during the quiet time intervention periods compared to the control conditions (7, 20).

Other clinician observation measurements for sleep in the ICU use visual cues such as eye closure and not moving to determine sleep duration (9), or asking the nurse one to two simple questions on the patient's sleep duration and sleep quality (retrospectively) (12, 13, 27). For example, Ibrahim and colleagues developed and used a nurse observation tool to record the total number of hours slept during the night and the day (9). Using this tool, the criteria for a patient to be considered asleep included eyes closed, lack of interaction with the environment, decreased motor activity, and lack of purposeful activity. The sleep observation tool by Ibrahim et al. was used as the primary outcome measure in one of the few studies where nurse observers were blinded to treatment condition. In a randomized double-blind placebo-controlled pilot trial (*n* = 32) to examine the effect of nocturnal melatonin on sleep duration in non-sedated ICU patients with tracheostomy, bedside nurses recorded the number of hours of observed sleep during the night (defined as 10 p.m.−6 a.m.) and during the day (defined as 6 a.m.−10 p.m.) (9). The frequency of the sleep observations was not discussed in the study methods, and there was insufficient discussion of measure properties, such as agreement among raters. There was no difference in observed nighttime sleep between the placebo group (243.4 min) and the melatonin group (240.0 min). Table 4 summarizes observation studies that measured sleep in critically ill patients.

**Table 4:** Observation of sleep in critically ill patients: performance, feasibility, responsiveness, missing data, and recommendations.

**Name of tool research design; sample**	**Performance (construct validity, criterion validity, reliability and consistency)**	**Feasibility**	**Responsiveness**	**Missing data**	**Comments/recommendations**
SOT (7, 78); Pre-post-test experimental; Neuro ICU *N* = 239	6 trained nurse observers IRR k = 0.93 Observations for at least 5 s, every 30 min, 8 times daily Construct validity—objectively measured light and sound levels were predictive of sleep (*P* < 0.001)	Yes	Yes, sleep, as measured by the SOT, changed in the expected direction. Patients were 1.6 times more likely to be asleep during the intervention compared to the control (*P* < 0.001	Not reported	Limitation: nurses were not blinded to intervention, or the light and sound measures, and lack of blinding may have affected responsiveness
Number of hours of observed sleep—by bedside nurses (9) RCT; *N* = 32 ICU patients with tracheostomy, not receiving sedation	Observational criteria for sleep were eyes closed, decreased motor activity, lack of interaction with the environment, and lack of purposeful activity. Validity of measure, training of observers, and IRR were not mentioned.	Yes	No. Placebo TST = 240 min (range 75–331.3) vs. Melatonin TST = 243.4 min (range 0–344)	Not reported	Recommend training of nurse observers, competency assessment, and assessment of IRR prior to, and quarterly during data collection
Investigator-developed single item ordinal scales: (1) hours slept, and (2) comparison with normal sleep Investigator-developed numerical rating scale (1–10) with anchors no sleep and slept well (13) Descriptive comparative; 4 multispecialty ICU's *N* = 82 patients and 82 nurses	Reliability and validity not discussed Items were derived and adapted from other sleep assessment tools Nurse researchers collected the data from the nurses and the patients Patients preferred the ordinal scales Association between nurse and patient, by scale: (1) G = 0.334, (2) G = 0.452; (3) G = 0.345, significance not reported	Yes	N/A. No intervention	Not reported	Strength: neither the nurse nor patient was aware of the other's rating There was an association between nurse and patient sleep assessment.
Hours slept and number of awakenings at the end of shift—by bedside nurses ^*^(12) Observational; Medical-Surgical ICU *N* = 12 (mechanically ventilated)	TST (hours)—Observation 5.35, PSG 3.10, Actigraphy 4.43 SEI—Observation 77.62, PSG 41.90, Actigraphy 61.30 Awakenings—Observation 8.5, PSG 40, Actigraphy 48.50	Yes	N/A, no intervention	Not reported	Nurses reported better sleep than measured by either PSG or actigraphy Nurses documented only about 1/5 of the awakenings shown by PSG and actigraphy Limitation: only 12 patients studied for only 1 night
SOT (78)—by bedside nurses (20) Experimental; Neuro ICU *N* = 50	The SOT was not compared with other measures in this study. Interrater reliability—nurse observations with the SOT were validated by comparing their results with those of the investigators prior to implementation of the study	Yes	Yes, the SOT was responsive. The results showed a change in sleep, in the expected direction, during the Quite Time intervention, compared to pre/post-test.	Not reported	Limitation: the nurses collecting the sleep data were unblinded to experimental condition
SOT (78)—by bedside nurses (24) Observational; 39 ICU units *N* = 539	The SOT was not compared with other measures in this study.	Unclear-large amount of missing data—reasons not discussed.	N/A No intervention	Sleep data missing in 163 (33%) of sample	Recommend using behavioral assessment of sleep combined with actigraphy, PSG, and other technologies to improve sleep/wake identification in objective measures.
Bedside nurses documented in the electronic medical record categorical data: no sleep, minimal sleep, moderate sleep, majority sleep, or slept all night (27) Descriptive; Medical ICU *n* = 151	Validity—RCSQ Questions 1–5 and nurse observation were significantly correlated (Spearman's rank correlation = 0.39–0.50, *P* < 0.001).	Yes	N/A, no intervention	Not reported	Recommend that nurses assess and document sleep quality and quantity as part of routine clinical care

* *See also Table 3. G, Gamma; IRR, interrater reliability; SEI, sleep efficiency index; SOT, Sleep Observation Tool; RCT, randomized clinical trial; TST, total sleep time*.

#### Summary of Strengths, Weaknesses, and Recommendations

Clinician observed sleep is particularly appealing for ICU patients who cannot provide information on perception of their sleep. Nurses are at the forefront of patient care, and they can provide important information on sleep while they are assessing other vital signs. Nurse observed sleep tools have the potential to be integrated into routine clinical practice, similar to pain assessments. In general, nurse observed sleep duration has shown good validity compared to other methods. For example, the SOT agreement with PSG-identified sleep was 81.9% (78). A study by Ritmala-Castren and colleagues in 20 general ICU patients reported that continuous nurse-observed patient sleep and wake state corresponded to PSG 2/3 of the time and sleep duration was very similar (observed sleep 6 h, 16 min; PSG sleep 6 h, 27 min). However, there was lack of correlation of nurse observation with other aspects of sleep including sleep latency, number of awakenings, and movements during sleep (79). Investigators should consider excluding sleep latency, number of awakenings, and movements as primary outcomes in clinical trials if they plan to use nurse observation as a sleep measure.

There are several methodological weaknesses in the literature and caveats regarding clinician observation of sleep. In studies to date, there is insufficient discussion of nurse observer training, agreement among the nurse observers, and discussion of any problems with missing data. These weaknesses may affect responsiveness in future clinical trials using these observational methods. Other potential limitations to structured observations are the potential to accidentally awaken the patient during the observation, blinding of nurses to intervention condition, and issues with feasibility such as insufficient nursing time for the observations. In other populations, trained research assistants often collect observational data. Investigators might consider research assistants, instead of nurses, for collecting observational data on sleep, especially when the nature of the intervention prevents blinding of the nurses.

### Patient Perception of Sleep Using Questionnaires

#### Background

Perception of sleep quality is an important dimension of sleep that may not be captured by objective measures. Decades of research have shown differences between sleep state perception and objectively measured sleep in a number of clinical sleep populations, most notably insomnia sufferers (80). A variety of patient completed questionnaires have been developed and used to assess perception of sleep in ICU and are covered by recent reviews (4, 79). In this review, we focus on the most commonly used tools, and emphasize the validated ones such as the Richard Campbell Sleep Questionnaire (RCSQ), and the tools that were developed more recently.

#### Results

The RCSQ is a five-item visual analog scale, measuring five domains of sleep, including sleep latency, sleep efficiency, sleep depth, number of awakenings, and overall sleep quality (2). The RCSQ is recommended by the clinical practice guideline for the management of sleep disruption in adult patients in the ICU (81). The RCSQ has demonstrated content validity, criterion validity against PSG, and internal consistency reliability with Cronbach's alpha of 0.90 (2), and has been used in well over 1,000 ICU patients. The RCSQ has been translated and validated in other languages, including versions in Arabic (82), Chinese (83), German (84), Japanese (85), and Portuguese (86). Although the RCSQ was developed as a self-assessment tool, some studies explored its patient-nurse interrater agreement. A slight to moderate correlation was observed between patient-completed and nurse-completed RCSQ scores (1), with the greatest divergence observed in female patients (63). A study in Australia reported a moderate agreement in patient-completed RCSQ and nurse-observed sleep (27).

The Verran Synder-Halpern Sleep Scale (VSH) is a visual analog scale (9–15 items, depending on version) that was originally developed and validated for measuring perception of sleep in healthy adults. It has subsequently been used and validated for sleep measurement in critically ill patients in several studies (14, 32, 34, 44).

Storti and colleagues developed a 9-item questionnaire, the Coronary Care Unit Questionnaire (CCUQ) to assess sleep in the coronary care unit (57). Although it was aimed to assess sleep quality, the majority of the items focused on sleep disruptors, such as “*did you find that the noise from the equipment of the intensive care interfered in the quality of your sleep*?” and “*did you find that your clinical condition interfered in the quality of your sleep*?” (57). A validation study (*n* = 99) showed adequate internal consistency reliability (Cronbach's alpha = 0.69) and significant correlation of the CCUQ total score with sleep efficiency from one night of PSG (*r* = 0.518).

In a recent study, Rood et al., conducted a large validation study (*n* = 194 Phase 1, and *n* = 1,603 in Phase 2) of a single item numeric rating scale (NRS—Sleep). The goal was to validate a method to assess sleep quality in everyday clinical practice. The NRS—Sleep significantly correlated with the RCSQ (*r* = 088, *p* < 0.01) and an optimal cut off value for good sleep was NRS > 5, with sensitivity 83%, and specificity 79% (62). A single rating scale can decrease the assessment burden and provide ongoing sleep quality data in clinical practice, but a 1-item tool may overlook important aspects of sleep such as depth, continuity, and latency that may be important to measure in clinical trials. Table 5 summarizes literature on critical care patients' perceptions of sleep using questionnaires.

**Table 5:** Patient questionnaires for measuring sleep in critically ill patients: performance, feasibility, responsiveness, missing data, and recommendations.

**Name of questionnaire**	**Measure performance (construct validity, criterion validity, reliability and consistency)**	**Feasibility/responsiveness/missing data**	**Comments/recommendations**
RCSQ (1, 2, 8, 17, 27, 31, 36, 46, 50, 53, 61–63)	The RCSQ is a 5-item questionnaire for patients to evaluate sleep depth, sleep latency (time to fall asleep), number of awakenings, sleep efficiency, and sleep quality. Each response is recorded on a 100 mm visual analog scale, with higher scores indicating better sleep and the total score representing overall perception of sleep quality. Internal consistency reliability was 0.90 and principal components factor analysis revealed a single factor (Eigenvalue = 3.61, percent variance 72.2). The RCSQ was significantly correlated with PSG variables, and total score accounted for about 33% of the variance in sleep efficiency index by PSG (*p* < 0.001).	Feasibility and missing data—Up to ½ of ICU patients cannot self-report their sleep due to delirium or sedation (17) which is a limitation of self-report measures that results in missing data or exclusion of a number of ICU patients from studies. Kamdar et al. (1) compared patient/nurse sleep assessments by RCSQ (*N* = 92 paired assessments). Bland Altman plots showed that nurses ratings were generally higher than patients and interrater reliability of patient-nurse pairs was slight to moderate. Responsiveness—The RCSQ significantly improved in response to a night-time noise and activities intervention (36). However, a large quality improvement study in ICU patients (*N* = 300) failed to show differences. Sleep was measured using the RCSQ by the patient (if able) or the nurse (if patient unable). There was no significant difference in sleep quality after introduction of a comprehensive quality improvement intervention.	ICU patients, total *N* = 1,243 (13 studies) The RCSQ is reliable and valid. Recommend that investigators report missing data.
VSH sleep scale (14, 32, 34, 44, 72)	The VSH sleep scale consists of visual analog items measuring perception of sleep the preceding night. Reliability coefficient was 0.82 (theta) in original 8-item scale, with 2-factors, disturbance and effectiveness; correlation with items on other validated sleep scales ranged from *r* = 0.22 to 0.74; 6 new items were added, resulting in a 14-item scale.	Feasibility—The revised VSH with 14–15 items may be too lengthy. Responsiveness—Yes, responsive in 3 studies, and in the same direction as other measures if reported. Missing data—None reported for VSH	ICU patients, total *N* = 212 (4 studies) A 15-item VSH scale was used in the Su study, Scotto and Hsu used the 8-item VSH scale, and Richardson used an 11-item VSH scale. Although the VSH was originally designed for healthy adults, reliability is adequate and validity supported for use in the ICU population. Recommend investigators report what version of the VSH they use, and the items included.
CCUQ (57)	The CCUQ was designed to evaluate sleep quality in the coronary care environment. It measures factors that impact sleep quality, such as bed quality, light, and noise and consists of 9 items, 1–5 points each, in Portuguese, with total scores ranging from 18 to 90 points, and higher scores indicating better sleep. In a validation study (*N* = 99, 67 males) internal consistency reliability was 0.69 (Cronbach's alpha) and the CCUQ total score was correlated with sleep efficiency from one night of PSG (*r* = 0.518, *p* < 0.001). Internal consistency reliability and criterion validity were both acceptable.	Feasibility—Yes, in this sample. Insufficient information on nursing time required to administer the tool. Responsiveness—N/A, no intervention. Missing data—None reported.	The CCUQ scale is unique because it measures factors that impact sleep in the ICU. It shows acceptable reliability and significant correlation with sleep efficiency as measured by PSG.
NRS—Sleep (62)	The NRS—Sleep is a single item numeric rating scale for ICU patients. The validation study was conducted in two phases. In the first phase, 468 ICU patients were enrolled, and 194 assessed sleep quality using the RCSQ and the NRS—Sleep. The NRS—Sleep significantly correlated with the RCSQ (*r* = 0.88, *p* < 0.01). Optimal cut-off value for good sleep was NRS > 5, with area under the curve of 0.81, and sensitivity of 83% and specificity of 79%. In the second phase 1,603 patients rated their sleep in 4,532 nights with the NRS—Sleep.	Feasibility and missing data—Over 50% of data were missing in phase 1 because of nursing and participant burden of completing both scales and the inability of ICU patients to self-report their sleep. Responsiveness—N/A, no intervention	The NRS is comparable to the RCSQ to assess sleep quality and is a feasible method to monitor sleep in everyday clinical practice. Recommend the NRS—Sleep be incorporated as part of routine ICU assessment. Recommend correlation of the NRS—Sleep by patients and by nurses to determine the validity of nurse ratings of patient sleep using the NRS—Sleep.
Investigator developed/modified tools (10, 13, 21, 28, 39, 41, 43, 45, 47, 55, 59, 60)	A variety of tools have been created or adapted, and used to collect information from ICU patients on sleep history, sleep disturbing factors, and sleep quality and quantity. Complete information on reliability and validity of these tools was lacking, but most clearly had content validity, and a few reported the development process and internal consistency reliability.	Feasibility—Yes, in these samples. Responsiveness—Unclear. Only (39, 41) measured patient perception of sleep related to an intervention (earplugs and eye masks). The sleep scales they used were ordinal. There was a significant improvement in sleep perception related to the intervention using an ordinal scale on 1 night, but no improvement in the other 4–5 nights. Missing data—Generally not reported. In the study by Van Rompaey 4 of 136 patients were not able to reply to the sleep questionnaire because of delirium.	ICU patients, total *N* = 1,013 (12 studies) This group of instruments requires further validation.

*CCUQ, Coronary Care Unit Questionnaire; PSG, polysomnography; RCSQ, Richards Campbell Sleep Questionnaire; NRS—Sleep, Numeric Rating Scale; VSH, Verran Synder-Halpern*.

#### Summary of Strengths, Weaknesses, and Recommendations

Patient perception of sleep quality is an important dimension for sleep clinicians and investigators to monitor, and it is of key importance to patients and their families. Similar to the findings from the recent review by Jeffs and Darbyshire (4), we found that a number of studies using questionnaires to measure perception of sleep in ICU patients reported no validity or reliability data for the tools they used. Future investigations should use valid assessment tools such as the RCSQ, VSH, CCUQ, or NRS—Sleep and provide justification for their choice of measurement(s). An inherent limitation for all self-assessment tools is that patients must to be alert, oriented, and able to respond and provide feedback. Up to ½ of critically ill patients may be unable to self-report their sleep quality. We recommend that investigators report missing data, and that self-report measures be augmented with observational methods and technology.

## Summary, Recommendations, and Future Directions

This review evaluated the measurement properties, feasibility, and responsiveness of existing instruments used to evaluate sleep in patients hospitalized in the ICU. An extensive search strategy resulted in 62 articles. We divided the instruments into 4 groups based on the dimensions of sleep they measured: (1) physiological sleep measured by polysomnography and other EEG-based methods, (2) actigraphy, (3) clinician observation, and (4) patient perception of sleep using questionnaires.

Sleep is multi-dimensional, composed of dimensions such as total sleep time, awakenings, expectations, global perceptions, movements, tiredness upon awakening, daytime energy, and function. Various measures of sleep in ICU patients exist, but they do not all measure exactly the same dimensions. Traditional measurement science specifies that while we expect correlation between dimensions of a construct, we would not expect the different dimensions to demonstrate 100% agreement. In general, we want to emphasize that PSG, other EEG-based methods, actigraphy, clinician observation, and patient perceptions provide complementary, but somewhat different information on sleep quality in critically ill patients. Given the multiple dimensions of sleep in critically ill patients, we highly recommend using multiple measures of sleep, especially in clinical trials. Clinical trialists should carefully consider sensitivity of outcome variables derived from each of the various sleep measurement methods, especially when choosing primary outcome variables for use in clinical trials. In general, awakenings are difficult to reliably capture in methods other than polysomnography.

Physiological sleep measured by PSG provides precise, objective information on sleep latency, sleep continuity, percent of sleep stages, sleep duration, and other objective sleep parameters. In general, it has excellent validity for recording physiological sleep. Although labor intensive, it is certainly feasible, as evidenced by the relatively large number of studies that have used PSG to study sleep in critically ill patients. The main drawback of PSG is reliability of scoring using standard AASM criteria due to the absence of stage N2 markers, polymorphic delta, burst suppression, use of sedating medications, electrical interference in the ICU, shivering, and other abnormalities or underlying illnesses. Several investigators have developed and validated alternative scoring methods for critically ill patients, but most studies to date have not used these new scoring methods. We recommend that investigators report and justify PSG scoring methods, and report scoring interrater reliability.

The traditional stage scoring of polysomnographic records provides basic information of sleep macroarchitecture, however, this method may be insufficient to detect sleep abnormalities in ICU patients who suffer from critical illnesses, external stimuli (e.g., psychotropic medications, ventilation, light, noise, treatment, and care), as well as potentially undiagnosed sleep disorders. Studies that have examined sleep microstructures in other populations provide insight into better understanding sleep abnormalities in ICU patients. For example, there can be significant arousal-related phasic events, even when the macrostructure of sleep appears to be normal (87). Another example, the cyclic alternating pattern (CAP), a periodicity dimension of NREM sleep that involves sleep microstructures such as high amplitude, slow EEG bursts, has been shown to be sensitive for identifying and quantifying sleep disturbances in subjects with decreased sleep quality (88, 89). In a well-designed case-control study comparing 78 untreated depressed patients with 18 age and sex-matched controls, there were no major differences in sleep macrostructure variables, but a significantly higher CAP (60% in depressed patients vs. 35% in controls) (90). In addition, examination of neural oscillations during sleep offers insights regarding neurophysiological functioning and network connectivity. In particular, sleep spindles analysis can facilitate our understanding of how brain activity during sleep is affected by sleep disorders (91). Therefore, in addition to the traditional macrostructure scoring, we recommend sleep microstructure analysis in future studies in critically ill patients.

Another challenge for observational PSG studies in critically ill patients is the selection of a control group for comparison. In all cases, we recommend that comparison groups should be matched on age, gender, and any other relevant factors. Investigators should also consider matching on relevant pre-morbid factors, such as reported sleep quality and overall health. Other important considerations, depending on the study aims, are mechanical ventilation and mode of ventilation, sleep promotion protocols, and sedating medications.

Compared to PSG, few studies of other portable EEG-based monitors have been conducted. While collecting data using EEG-based monitors is less labor-intensive than PSG, their validity in the ICU setting requires further testing. We recommend, when possible, they be used along with other methods to provide valuable validity data.

In this review, we identified only 9 actigraphy studies in critically ill patients, most had small sample sizes, only about ½ evaluated measurement properties, and reliability of PSG scoring was infrequently mentioned. Overall, actigraphy tended toward more total sleep time, higher sleep efficiency, and more frequent nighttime awakenings compared to PSG, and more overall awakenings compared to nurse assessment and patient questionnaires. Additional research on actigraphy in critically ill patients is needed with larger sample sizes, longer durations, and specific sensors and settings for low mobility states. In addition, the exclusion criteria for reliable actigraphy in the ICU population requires further discussion and consensus.

Systematic clinician observation for sleep and wake states by nurses or other trained personnel is a good choice, especially for those ICU patients who are unable to self-report. The clinician observation method requires that coding of sleep or wake data is based on the presence or absence of specific behaviors, and that the data collectors have been trained and verified as competent. Similar to PSG scoring, interrater reliability (consistency between data collectors on coding the behavior) is important. We recommend that clinicians and investigators use the SOT because it was excellent agreement with PSG-identified sleep. Nurse observed sleep tools have the potential to be integrated into routine clinical practice, similar to pain assessments. Unfortunately, in studies to date, there is insufficient attention paid to nurse observer training, agreement among the nurse observers, and discussion of missing data. Other potential limitations of systematic observation methods are the potential to accidentally awaken the patient during the observation and issues with feasibility such as insufficient nursing time. In clinical trials, it is important that those collecting the outcome data are blinded to group assignment, which may preclude nurses from collecting observational data in some clinical trials. Investigators might consider research assistants for sleep observations when the nature of the intervention prevents blinding.

Sleep questionnaires measure patients' perceptions of their sleep quality. A limitation for all self-assessment tools is that patients have to be alert, oriented, and able to respond and provide feedback. However, perception is an important dimension of sleep that may not be totally captured by other objective measures. Perception of ICU sleep is influenced by many factors, including usual home sleep quality and patterns, and expectations. We recommend the RCSQ for sleep assessment in ICU patients, based on its reliability and validity, and feasibility. The RCSQ also has the advantage of several validated translated versions for non-English speakers. We recommend that the NRS—Sleep be incorporated into routine ICU assessment.

Future directions for ICU sleep research might include new methods for identifying sleep using machine learning to analyze the multitude of data already continuously collected in ICUs, such as heart rate, blood pressure, and oxygen desaturation to identify sleep and wake, and perhaps NREM and REM sleep. Accuracy of these methods might be improved by adding additional sleep-specific devices, such as the EOG and EMG. Another idea to improve the feasibility and accuracy of observation of sleep and wake, or replace it, is face recognition technology.

In conclusion, there is ample evidence that sleep deprivation during the ICU stay has negative short-term effects, and serious lasting consequences that are of key importance to patients. Measuring the impact of interventions to improve sleep and prevent sleep deprivation requires reliable and valid sleep measures, and investigators have made good progress developing, testing, and applying these measures in the ICU. We recommend future large, multi-site intervention studies that measure multiple dimensions of sleep, and provide additional evidence on instrument reliability, validity, feasibility, and responsiveness. We also encourage testing new technologies to augment existing measures to improve their feasibility and accuracy.

## Author Contributions

KR, Y-yW, JJ, and LY planned the manuscript. KR, Y-yW, JJ, and LY wrote the manuscript and carried out the subsequent revisions. Y-yW provided primary contributions to polysomnography part. JJ provided primary contributions to actigraphy part. LY provided primary contributions to subjective measurements part. KR provided substantial contributions to the whole process of manuscripts writing and revisions. All the co-authors provided substantial contributions to the first manuscript draft and subsequent revised versions.

## Conflict of Interest

The authors declare that the research was conducted in the absence of any commercial or financial relationships that could be construed as a potential conflict of interest.
